# Functionalized Gold Nanoparticles for the Detection of C-Reactive Protein

**DOI:** 10.3390/nano8040200

**Published:** 2018-03-28

**Authors:** Maria António, João Nogueira, Rui Vitorino, Ana L. Daniel-da-Silva

**Affiliations:** 1 CICECO-Aveiro Institute of Materials, Department of Chemistry, University of Aveiro, 3810-193 Aveiro, Portugal; maantonio@ua.pt (M.A.); jh.nogueira@ua.pt (J.N.); 2 iBiMED-Institute of Biomedicine, Department of Medical Sciences, University of Aveiro, 3810-193 Aveiro, Portugal

**Keywords:** gold nanoparticles, surface functionalization, C-reactive protein, biodetection

## Abstract

C-reactive protein (CRP) is a very important biomarker of infection and inflammation for a number of diseases. Routine CRP measurements with high sensitivity and reliability are highly relevant to the assessment of states of inflammation and the efficacy of treatment intervention, and require the development of very sensitive, selective, fast, robust and reproducible assays. Gold nanoparticles (Au NPs) are distinguished for their unique electrical and optical properties and the ability to conjugate with biomolecules. Au NP-based probes have attracted considerable attention in the last decade in the analysis of biological samples due to their simplicity, high sensitivity and selectivity. Thus, this article aims to be a critical and constructive analysis of the literature of the last three years regarding the advances made in the development of bioanalytical assays based on gold nanoparticles for the in vitro detection and quantification of C-reactive protein from biological samples. Current methods for Au NP synthesis and the strategies for surface modification aiming at selectivity towards CRP are highlighted.

## 1. Introduction

Human C-reactive protein (CRP) is a major acute-phase reactant protein produced in the liver and is present in blood plasma [1,2]. CRP is used for standard clinical practice [3] and is one of the most well-known biomarkers in cardiovascular diseases [4]. High-sensitivity CRP (hs-CRP) assays have been involved in the detection of small variations in CRP concentrations [5]. The American Heart Association and the Center for Disease Control and Prevention determined that cardiovascular diseases risk differ depending on CRP concentration. More specifically, CRP levels < 1 mg·L^−1^ show a low systemic inflammatory status, which is desirable; levels between 1 and 3 mg·L^−1^ reflect moderate vascular risk; whereas levels > 3 mg·L^−1^ indicate higher vascular risk. For values of hs-CRP superior to 10 mg·L^−1^, a transient infectious process or other acute-phase response could be occurring and this result needs to be revaluated in 2 to 3 weeks [6].

The analytical methods to assess hs-CRP levels should be sensitive, selective, fast and trustworthy using the lowest sample volume (sensitive analysis of CRP levels in biological samples) [7]. Ideally the method should also be easy to perform and cheap in order to be widely used in clinical diagnostics for the prevention of severe inflammatory states [8]. The in vitro detection of CRP has been studied through numerous techniques such as enzyme-linked immunosorbent assay (ELISA), electrochemical assay, surface plasmon resonance assay, fluorescence assay and chemiluminescence assay [7]. These methods should have the ability to detect CRP in concentrations below pg·mL^−1^ for an accurate measurement of CRP in the population. Among the techniques mentioned, that which is most used for the in vitro detection of CRP is ELISA. ELISA assays provide good sensitivity (pg·mL^−1^ or fg·mL^−1^) [9] and are available in the form of a kit test [10]. However, this technique presents several drawbacks, including: large quantities of sample are needed; the use of antibodies which usually have low stability and need specific chemical medium conditions; high production costs [9]; long reaction time [11]; the need for trained professionals and expensive reagents [12]; and relatively high false-positive rates owing to non-specific binding [10]. In addition, sandwich immunoassays frequently lead to false-negative results at high concentrations of the analyte owing to the “Hook effect” [13]. These disadvantages triggered the need to develop novel strategies for CRP detection.

**Figure 1:**
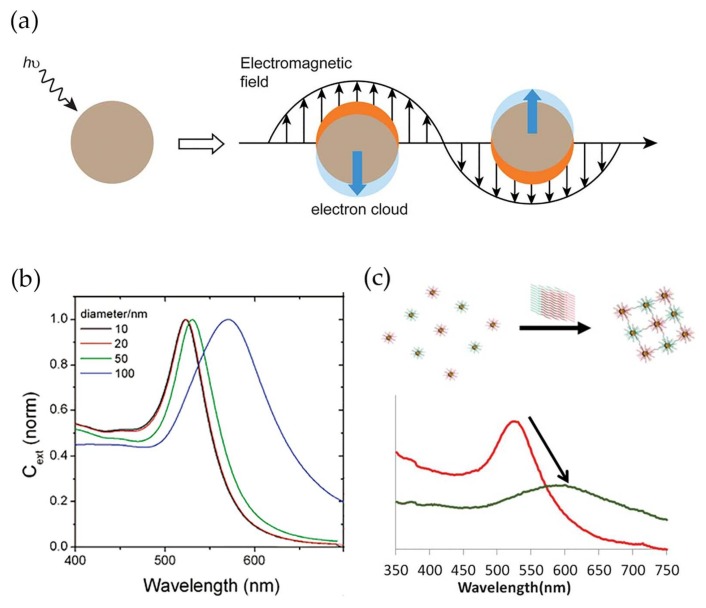
(**a**) Schematic illustration of localized surface plasmon resonance effect. Conduction electrons oscillate across the metal nanoparticle owing to the electromagnetic field of the incident light. Reproduced with permission from ref. [30]. Copyright The Royal Society of Chemistry, 2012; (**b**) Dependence of localized surface plasmon resonance (LSRP) band wavelength to average diameter of spheroidal gold nanoparticles. Reproduced with permission from ref. [23]. Copyright American Chemical Society, 2006; (**c**) Variation in LSPR band between modified gold nanoparticles (red) and modified gold nanoparticles conjugating after recognizing the target (green). Reproduced with permission from ref. [31]. Copyright The Royal Society of Chemistry, 2013.

Gold nanoparticles (Au NPs) present high chemical stability, easy and reproducible preparation and surface modification methods, shape and size controllability, and low-toxicity, properties that have attracted substantial attention for several biological applications including in vitro detection and diagnostics [14,15,16,17]. In addition, gold nanoparticles display high surface-to-volume ratios, which contribute to very high loading capacities and lead to improved sensitivity of the analytical system [18,19,20]. Most relevant for bio-sensing applications are the unique electrical and optical properties of gold nanoparticles, namely the noticeable band in the optical spectrum, known as localized surface plasmon resonance (LSPR) band. This band arises due to the scattering and absorption of photons as a result of the resonant coherent collective oscillation of the surface electrons in gold nanoparticles along with the oscillating electromagnetic field of the incident light with a specific frequency (Figure 1a) [21]. The wavelength of the LSPR band varies with the particle size, morphology and nature of the surrounding medium [22]. In spherical NPs, an increase of the average diameter from 10 to 100 nm leads to a red shift of 47 nm (Figure 1b) [23]. The optical response also depends on the dielectric function of the surrounding medium. Thus, an increase of the dielectric constant of the medium causes the shift of the LSPR band to higher wavelengths due to the weakening of the restoring force caused by the formation of polarization charges at the interface with the dielectric medium [24]. Furthermore, when the gold nanoparticles are within distances comparable to their diameter, the LSPR band displays a red-shift combined with enlargement, owing to plasmonic coupling. Due to its sensitivity, the LSPR band is a valuable feature in the detection method for bio-specific interaction analysis and biomolecular interaction assays (Figure 1c) [25,26,27]. In addition, Au NPs and noble nanoscale metals, in the presence of local electromagnetic fields, could interact with fluorophores through coupling their plasmonic scattering modes to increase their fluorescence intensity. This could improve both excitation and emission fluorescence processes [28] in immunoassays [29].

In this review, we present an overview of the latest developments on high-sensitivity assays for in vitro detection of CRP based on gold nanoparticles. Emphasis was given to strategies for surface modification and bioconjugation of gold nanoparticles, which are crucial to impart specificity towards CRP and attain high sensitivity. Emerging trends of bioconjugation beyond conventional antibodies such as aptamers are discussed. Examples of electrochemical and LSPR-based assays recently developed are described. Furthermore, the latest advances on the application of gold nanoparticles in point-of-care (POC) assays are also reviewed.

## 2. Synthesis and Modification of Gold Nanoparticles (Au NPs)

As described in the previous section, the optical properties of gold nanoparticles are strongly dependent on the particle size and shape. Thus, a number of synthetic strategies have been developed for the synthesis of gold nanoparticles with uniform morphology, narrow size distribution and tailored properties, as extensively reviewed elsewhere [30,32,33,34].

### 2.1. Synthesis of Au NPs

**Figure 2:**
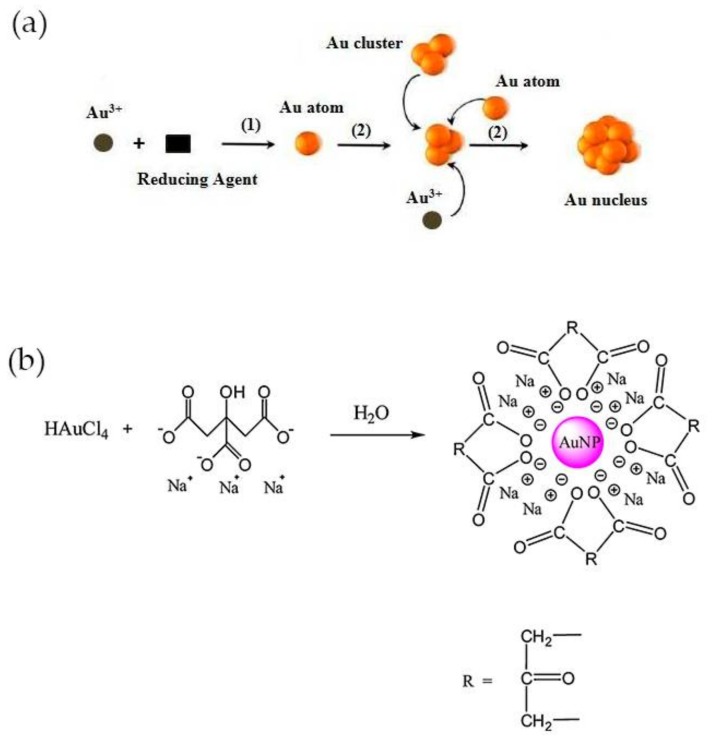
(**a**) Process of nucleation for synthesis of Au NPs by chemical reduction method. Reproduced with permission from ref. [35]. Copyright Elsevier Ldt, 2010; (**b**) Representation of Turkevich method. Reproduced with permission from ref. [33]. Copyright Elsevier B. V., 2012.

Examples of methods for the synthesis of colloidal gold nanoparticles include wet chemical processes like the chemical reduction of Au salts, electrochemical pathways, and the decomposition of organometallic compounds. From these methods, chemical reduction is the easiest to prepare stable colloidal Au NPs with desirable sizes and shapes [35]. Typically, it involves two-steps, the nucleation and successive growth of the nanoparticles. The formation of nuclei occurs by reduction of Au(III) species with a reducing agent (1) and the collision between ions, atoms and clusters (2) as represented in Figure 2a [35]. The size and shape-controlled growth of the Au nanoparticles depends on the reaction conditions such as the concentration of reactants, temperature and duration of growth stage. The most common shape of these nanoparticles is spherical, owing to isotropic growth on the surface of Au nuclei or particles. Even so, Au NPs can be synthesized into triangle, rod, cube, star, wire, and flower shapes if anisotropic growth is promoted [35,36]. The earliest-reported methods for the synthesis of spheroidal nanoparticles were developed by Faraday, Turkevich and Brust [37,38]. The two-step synthesis of colloidal gold was proposed by Faraday, in 1857 and involved the reduction of tetrachloroaurate ions (AuCl_4_^−^) by phosphorus in carbon disulphide [39]. Conversely, Turkevich developed in 1951 a single-phase aqueous method based on the reduction of a gold salt by citrate [40].

The Turkevich method is the one most commonly used for preparing gold nanoparticles for CRP detection. In this method, trisodium citrate is used both as reducing and stabilizing agent and is added to chloroauric acid (HAuCl_4_) boiling solution under vigorous stirring. A red-wine solution is obtained at the end of the reaction, owing to the formation of Au NPs with an average diameter of around 20 nm, with a negatively charged surface and electrostatically stabilized by citrate ions (Figure 2b) [40]. The size of the nanoparticles can be adjusted by varying the citrate/Au(III) molar ratio. At high ratio values, the nucleation is faster, dominant and leads to the formation of smaller NPs, while larger particles, up to 150 nm, may be obtained at lower ratios [41]. The method was further improved later by Frens, which produced almost spherical particles over a tunable range of sizes [42]. Smaller (<10 nm) and monodisperse Au NPs can be prepared by simply reversing the order of addition of the reactants. Thus, the addition of the HAuCl_4_ solution to the citrate solution favors the nucleation and stabilization of Au NPs [43]. Despite the electrostatic stabilization, citrate-stabilized particles obtained using the Turkevich method can aggregate during storage or subsequent functionalization steps. Large molecules such as surfactants and mercaptoalkanoic acids can be used to functionalize the surface and prevent the aggregation of Au NPs [30].

Smaller spherical Au nanoparticles, with an average size of 2 nm and narrow size distribution can be obtained using sodium borohydride (NaBH_4_), which is a stronger reducing agent, following the Brust–Schiffrin method, a two-phase method that yields thiolate-stabilized Au NPs in an organic phase [37]. However, the resulting Au NPs are not dispersible in water [44,45] nor in common buffers which limits its applications in the biomedical field. To confer water compatibility the surface of Au NPs must be further modified with hydrophilic capping agents such as sugars [46], polymers [47] and peptides [48,49,50]. Nevertheless, small Au NPs are less attractive for biodetection, because they exhibit low extinction coefficients when compared with larger-size gold nanoparticles [51,52]. Gold nanoparticles with spherical or spheroidal shape are the most commonly used in SPR–based biosensors [27]. Although nanorods are more sensitive to the refractive index changes than nanospheres [25], they require more complicated surface chemistry modifications routes for biosensing than in the case of spherical nanoparticles synthetized via the Turkevich method [53].

### 2.2. Surface Modification of Au NPs for C-Reactive Protein (CRP) Detection

Sensitivity in biosensing depends on a proper surface modification of Au NPs that should provide both adequate functionality and colloidal stabilization [19]. The selection of the Au NP modification strategy and the binding affinity of the interacting biomolecule with CRP influences the sensitiveness of the system developed.

Numerous strategies have been described in literature for the surface modification of spherical Au NPs with antibodies, aptamers or phosphocholine groups for CRP detection [12,13,54,55,56,57,58,59,60]. These strategies are summarized in Table 1.

#### 2.2.1. Bioconjugation with Antibodies

Gold nanoparticles can be conjugated with antibodies either via non-covalent or covalent immobilization strategies [57,61]. In the first approach, antibodies are immobilized at the surface of Au NPs through electrostatic and/or hydrophobic interactions [13,54,55,56]. Electrostatic interactions are possible between the positively charged side chains or protonated N-terminal groups of aminoacids and the negatively charged surface of Au NPs. Hydrophobic interactions take place between the hydrophobic parts of the antibody and the gold metal surface [62]. 

The non-covalent immobilization of the antibodies can be affected, for instance, by changes in the medium conditions. In order to obtain more robust biosensors it might be necessary to covalently bind the antibody to the modified surface of Au nanoparticles. The most common strategy is the modification of the gold surface with carboxylic acid groups using, for example, 3-mercaptopropionic acid (MPA), followed by reaction with primary amine groups of the CRP antibody for specific covalent binding via carbodiimide coupling and formation of an amide bond [12,57]. *N*-(3-Dimethylaminopropyl)-*N*′-ethylcarbodiimide (EDC) and *N*-Hydroxysuccinimide (NHS) have been used to enhance –COOH reactivity towards –NH_2_ groups in a CRP antibody. Frequently, after antibody binding, the “free” gold surface is modified with thiolate polyethylene glycol or bovine serum albumin (BSA) to avoid non-specific interactions. Other options are available for covalent immobilizations such as the formation of avidin-biotin complexes [62,63].

#### 2.2.2. Surface Modification with Phosphorylcholine

**Figure 3:**
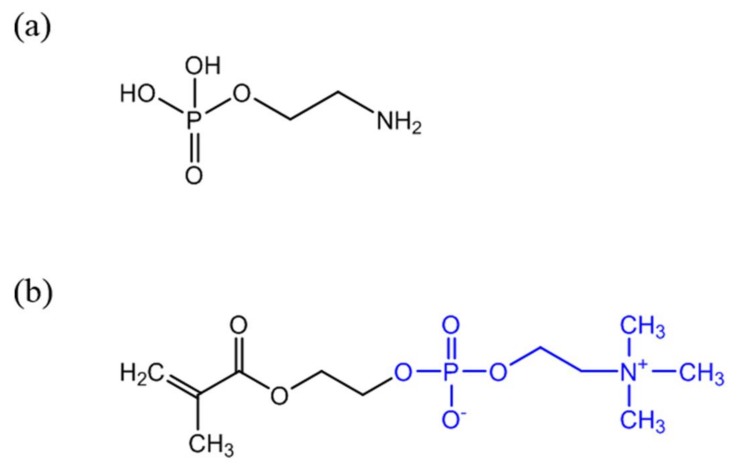
Chemical structure of PEA (**a**) and 2-methacryloyloxyethyl phosphorylcholine (MPC) containing PC functionality (**b**) (PC group represented in blue).

Antibodies (anti-CRP) are expensive and unstable, and to overcome these drawbacks alternative strategies to confer CRP recognition to Au NPs have been investigated. It is well known that in the human body CRP naturally binds to phosphorylcholine groups (PC) on the cell membranes of dead or dying cells, mediated by calcium ions. Phenylalanine (Phe)-66 and glutamic acid (Glu)-81 are the two key residues participating in the binding of CRP to PC [64]. Thus, an attractive and less expensive alternative to antibodies is the covalent grafting of compounds with PC functionality onto the surface of Au NPs. For example, *O*-phosphorylethanolamine (PEA) (Figure 3a) was covalently linked to Au NPs with surfaces previously modified with 16-mercaptohexadecanoic acid (MHDA). The reaction followed the carbodiimide chemistry using EDC to activate the carboxylic acid groups of MHDA and promote the reaction with amine groups of PEA [58]. Non-covalent immobilization of PEA on Au NPs for CRP detection has been also reported [65]. Recently, a 2-methacryloyloxyethyl phosphorylcholine (MPC) polymer (Figure 3b) has been shown to be a good indicator for detecting CRP [63] and previous studies suggested that it suppresses non-specific interactions with plasma proteins [66]. Subsequently, several MPC derived polymers have been used for Au NPs surface modification aiming CRP sensing. For example poly(2-methacryloyloxyethylphosphorylcholine) (PMPC) was grafted to the surface of Au NPs using surface-initiated atom transfer radical polymerization and the resulting polymer layer provided high-sensitive CRP recognition to Au NPs [67]. Iwasaki and coworkers [59] developed a distinct strategy that involved the simultaneous Au NP synthesis and surface modification for CRP recognition, using a thiol-terminated block copolymer, poly(2-methacryloyloxyethyl phosphorylcholine)-b-poly(*N*-methacryloyl-(l)-tyrosine methylester) (PMPC-b-PMAT). By simply adding HAuCl_4_ aqueous solution to PMPC-b-PMAT in alkaline conditions, spherical Au NPs with sizes of 3–13 nm were successfully prepared owing to the reducing action of tyrosine. On the other hand, the phosphorylcholine moieties provided specificity towards CRP. In addition, the resulting conjugated Au NPs exhibited excellent colloidal stability at high salt concentration and over a wide pH range because of the effective steric stabilization provided by the co-polymer PMPC-b-PMAT.

#### 2.2.3. Bioconjugation with Aptamers

Less explored is the surface modification of Au NPs with aptamers. Aptamers are target specific ssDNA, RNA or peptide sequences generated by a randomized library of molecules [68]. The first aptamer was reported in 1990, and since then it has attracted increased attention in several applications for analytical methods [69,70]. The aptamer-generation process includes three distinct stages: complex formation, separation and amplification. Systematic evolution of ligands by exponential enrichment, Systematic Evolution of Ligands by Exponential Enrichment (SELEX), generates aptamers by incubation of the initial target, and library molecules (a random single-stranded DNA or RNA pool) are used under appropriate conditions to promote complex formation. Afterwards, uncomplexed molecules are separated and bound molecules are amplified for a subsequent round of selection [68,71]. Compared to antibodies, aptamers present a number of advantages, such as easier preparation and modification, low cost, minimum batch-to-batch variation, stability and non-immunogenicity. Owing to these advantages, aptamers are emerging in numerous applications such as therapeutics and diagnoses [70,71,72]. 

Aptamers can be easily modified or linked to other molecules and have high specificity and potential to be used in CRP-detection assays either by themselves [73] or combined with antibodies [56,60] or organic dyes [69]. Nevertheless, reports of aptamer-based assays for CRP detection are still scarce.

Thiol-modified aptamers have been used for surface modification of Au NPs [56] and Au films for CRP detection [60]. In both systems the thiol group of the aptamer interacted with gold surface and established a strong interaction via the Au–S bond.

## 3. CRP-Detection Assays Using Gold Nanoparticles

In this section the most representative examples of recent Au NP-based CRP-detection assays are illustrated, categorized by a signal transducer including electrical, LSPR and their integration with POC systems. The most relevant characteristics are summarized in Table 2.

### 3.1. Electrochemical Detection

Electrochemical assays can use distinct procedures for the detection of CRP, namely amperometric, potentiometric and electrochemical impedance spectroscopy (EIS) [7]. Gold nanoparticles have been used mostly in EIS-based assays to provide high-density antibody immobilization and enhancement of electron transfer, thus improving the sensitivity in CRP detection [12,18]. EIS-based assays monitor the variations in capacitance and charge-transfer resistance related to the binding materials on the electrode surface, requiring no labelling [7]. Herein we describe the advances in EIS-based systems using gold nanoparticles.

**Figure 4:**
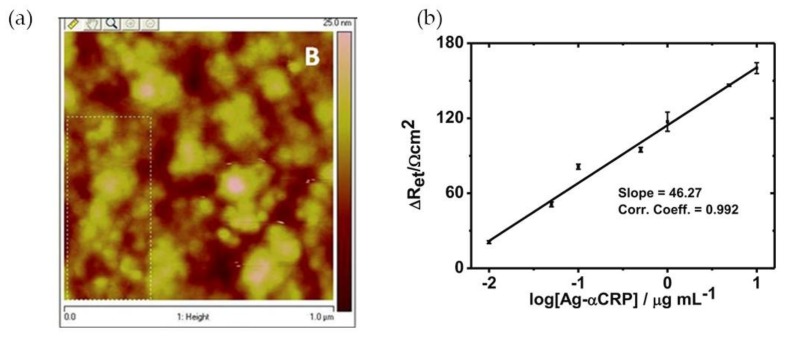
(**a**) Atomic force microscopy (AFM) image of Ab-αCRP/Au(MPA)-PPy-glass electrode; (**b**) Illustration of calibration curve of bioelectrode. Reproduced with permission from ref. [12]. Copyright Springer Science+Business Media New York, 2014.

Mishra and colleagues [12] have reported a CRP EIS-based sensor comprising an Au(MPA)-polypyrrole (PPy) nanocomposite film, with 3-mercaptopropionic acid-capped gold nanoparticles, of controlled thickness, electrochemically deposited onto an indium tin oxide-coated glass plate. The polypyrrole is a conducting polymer that permits a facile electronic charge flow across the sensing region. The incorporation of Au(MPA) nanoparticles provided free pendant carboxylic groups which were used for site-specific covalent binding of protein antibody (Ab-αCRP) via carbodiimide coupling. Atomic force microscopy (AFM) analysis of the film’s surface showed globular structures that can be ascribed to a non-compact assembly of Ab-αCRP molecules (Figure 4a). Gold nanoparticles also enhanced the conductivity of the nanocomposite film due to the metal’s electrical conductivity and improved the response of the sensor owing to high antibody loading and efficient covalent bonding to Au NPs. For CRP quantification EI spectra were acquired at low frequencies (<10 Hz) and an increase in charge transfer resistance (*R*_et_) indicated the presence of CRP antigen (Ag-αCRP). A linear relationship between the increased ∆*R*_et_ values and the logarithmic value of protein antigen was found in the range of 10 ng·mL^−1^ to 10 μg·mL^−1^ Ag-αCRP (phosphate-buffered saline (PBS), pH 7.4). The biosensor showed good *R*_et_ sensitivity (46.27 Ω cm^2^/decade) of Ag-αCRP (Figure 4b). The CRP-detection limit of this system was 19.38 ng·mL^−1^. 

A similar strategy was followed by Zhang et al. [18] who developed a label-free immunoassay composed by glassy carbon electrode (GCE) coated with a high conductive substrate of molybdenum disulfide-polyaniline (MoS_2_-PANI) nanocomposite and decorated with gold nanoparticles for CRP detection. The anti-CRP antibodies were dropped onto the gold nanoparticles, without any previous surface modification, which indicates non-covalent bonding of the antibodies at the surface of Au NPs. Cyclic voltammetry studies showed that the peak current decreased and the impedance increased in the presence of CRP due to the formation of the antibody-antigen complex. The optimal conditions for the detection of CRP were found to be 30 μg·mL^−1^ anti-CRP and an incubation time with CRP of 50 min. In the trial, CRP was added after anti-CRP reacted for 1h with the modified GCE surface. The detection limit obtained was 40 pg·mL^−1^ and the method has shown to be reproducible and stable. 

**Figure 5:**
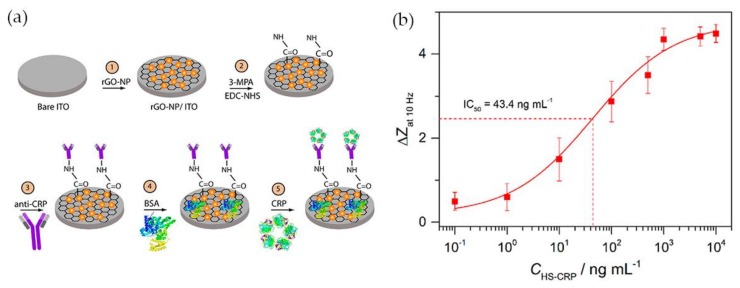
(**a**) Schematic illustration of anti-CRP/MPA/rGO-Au NP/ITO biosensor; (**b**) Calibration curve of CRP impedance biosensor in human serum spiked with CRP (HS-CRP). Reproduced with permission from ref. [57]. Copyright Elsevier, 2015.

Having in mind the excellent electrical conductivity of reduced graphene oxide (rGO), Yagati and co-workers [57] have developed a biosensor composed of rGO-Au NPs hybrid structures deposited on indium tin oxide (ITO) microdisk electrode array (MDEA) chips. The rGO-Au nanocomposite was synthesized by electrodeposition using a cyclic voltammetric technique with a homogeneous mixture of rGO and tetrachlorauric acid. ITO surface analysis confirmed the formation of spheroidal Au NPs 50 nm size, homogeneously distributed and stacked into rGO film without aggregation. For CRP detection rGO-Au NP MDEAs were incubated with MPA followed by covalent binding of antibody using EDC/NHS. The surface was then treated with bovine serum albumin (BSA) to block nonspecific adsorption (Figure 5a). The system was tested for CRP detection in human serum (HS) and the impedance (*Z*) was measured in CRP spiked serum samples of various concentrations (HS-CRP). The normalized impedance change at 10 Hz (Δ*Z*) calculated through Equation (1) increased with increasing CRP concentration (Figure 5b).

Δ*Z* = (|*Z*|_HS-antigen_ − |*Z*|_anti-CRP Ab_)/|Z|_anti-CRP Ab_ (1)

In human serum, the CRP concentration exhibited a linear relationship with the normalized impedance within the concentration range 2 ng·mL^−1^ to 1 μg·mL^−1^ and the detection limit was 0.08 ng·mL^−1^. The rGO-Au based EIS system was shown to allow measuring of the CRP concentration in human serum with high sensitivity and reliability, which demonstrates the potential of the platform for application in CRP biosensing.

**Figure 6:**
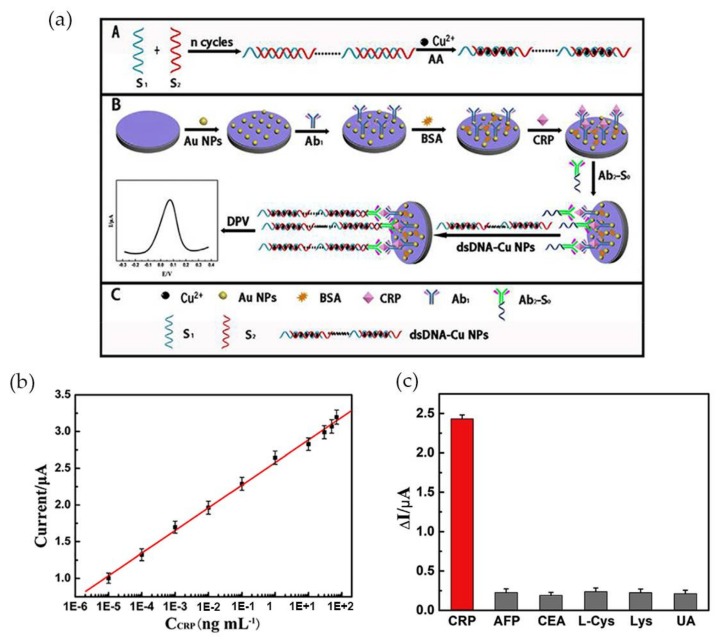
(**a**) Schematic illustration of the preparation of the electrochemical immunosensor; (**b**) Calibration curve of peak current using variable CRP concentrations and 10% of serum; (**c**) Selectivity of CRP testing sensor for 0.1 ng·mL^−1^ CRP and 10 ng·mL^−1^ of the interfering components alpha-fetoprotein (AFP), carcinoembryonic antigen (CEA), l-Cystein (l-Cys), lysine and uric acid (UA), tested individually. Reproduced with permission from ref. [74]. Copyright Elsevier Inc., 2017.

More recently Zhang et al. [74] developed an ultrasensitive Au NP-based biosensor for electrochemical CRP detection, using the hybridization chain reaction (HCR) technique to amplify the response signal. The biosensor was composed by three different parts: the primary antibodies (anti-CRP) immobilized in Au NPs (Ab_1_), a bioconjugate of a secondary antibody with a primer with a specific sequence complementary to DNA concatemers (Ab_2_-S_0_) and, the DNA concatemers. Ab_2_ was covalently linked to the primer, via carbodiimide chemistry (EDC/NHS) and Cu NPs, used as a signal tag, were synthesized in site, intercalated with DNA concatemers (dsDNA-Cu NPs) and then linked to the sandwich-type structure via a hybridization reaction as Figure 6a illustrates.

The analyte CRP interacted with Ab_1_-Au NPs at the electrode, and subsequently with the conjugate Ab_2_-S_0_, while dsDNA-Cu NPs were used as signal probe. The electrochemical signal emitted by the Cu NPs was monitored by differential pulse voltammetry (DPV) in the presence of CRP. Increasing CRP concentrations led to an increase in the electrochemical signal. The peak current was higher at pH 6.0, for a CRP concentration of 0.1 ng·mL^−1^ added and at an optimized incubation time of 40 min. The sensor exhibited wide linear range for CRP concentration between 1.0 fg·mL^−1^ and 100 ng·mL^−1^ and high sensitivity with a detection limit of 0.33 fg·mL^−1^ (Figure 6b). Tests performed with 10% of serum revealed a detection range from 10 fg·mL^−1^ to 70 ng·mL^−1^ and a correlation coefficient of 0.9986. It was also proved that the system was specific for CRP by adding 10 ng·mL^−1^ of alpha-fetoprotein (AFP), carcinoembryonic antigen (CEA), l-Cystein (l-Cys), Lysine and uric acid (UA) to 0.1 ng·mL^−1^ of CRP, with the interference of each compound less than 8% (Figure 6c). 

**Figure 7:**
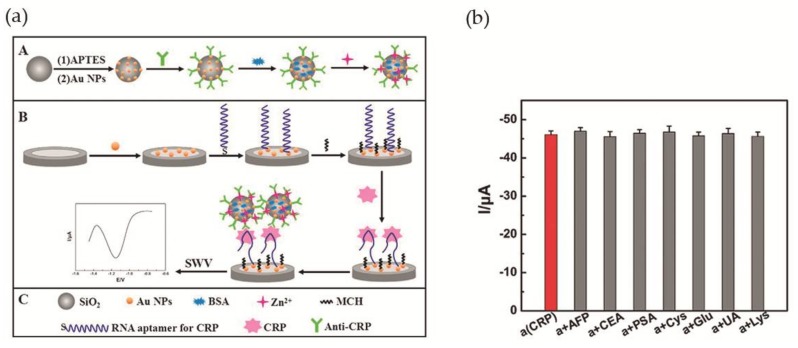
(**a**) Illustration of the assembled CRP aptasensor. A—Modification of Si MSs with Au NPs, anti-CRP, BSA and zinc ions. B—Modification of bare GCE with Au NPs, RNA aptamer and 6-mercapto-1-hexanol (MCH). Aptasensor detecting CRP and square wave voltammetry (SWV) record of the signal; (**b**) Changes in peak current during CRP detection in the presence of interfering compounds. Reproduced with permission from ref. [56]. Copyright Elsevier B. V., 2017.

Wang et al. [56] accomplished a RNA aptamer-based electrochemical CRP aptasensor. This system was composed of amorphous silica (SiO_2_) microspheres (Si MSs) loaded with zinc ions. Concisely, after silica microspheres synthesis, amino-Si MSs were added to an Au NPs solution, and the Au NPs assembled to the surface via Au-N bond. Finally, anti-CRP was added to Au NPs@Si MSs, as well as, 1% of BSA solution and Zn(II) aqueous solution. The Zn^2+^ was immobilized on the surface of the particles. The second part of the aptasensor was a bare GCE modified with Au NPs (Au NPs/GCE) that was immersed in a RNA aptamer solution, for interaction with Au NPs via Au-S bonds. Figure 7a illustrates the assembled aptasensor. Cyclic voltammetry (CV) and EIS measurements were used to confirm the proper construction of the aptasensor. In addition, ultravoilet-visible (UV-vis), scanning electron microscopy (SEM) and energy-dispersive X-ray spectroscopy (EDX) were used for the same propose. For optimization of the experimental conditions and in order to obtain a more sensitive system, the optimal concentration of aptamer was adjusted to 0.1 μM, the pH to 5.5 and the time for immunoreaction to 50 min. Square wave voltammetry was used to investigate the analytical performance of the aptasensor. It was observed that, as CRP concentration increases, the signal intensity of Zn^2+^ increases as well. The detection limit was determined as 0.0017 ng·mL^−1^, in a linear range of 0.005–125 ng·mL^−1^. Selectivity was evaluated by addition of 10 ng·mL^−1^ of AFP, CEA, prostate specific antigen (PSA), Cys, glucose (Glu); UA and Lys to 0.1 ng·mL^−1^ of CRP, demonstrating a good selectivity (Figure 7b). The aptasensor was tested for human serum samples and compared with immunofluorescence method used as reference. The difference between methods was less than 4.2%. Also, the possibility of recovering the sample after the aptasensor was used for CRP detection was explored, with 96.1–108.1% (standard derivation of 1.77–5.23%) of the sample recovered.

### 3.2. Localized Surface Plasmon Resonance (LSPR)-Based Detection

The colloids of gold nanoparticles exhibit variable color that depends on the particle size [23]. Changes in the colloidal stability from non-aggregate to aggregate state lead to measurable variations in the LSPR band wavelength and intensity [59,67]. An LSPR-based nanosensing system has multiple advantages, such as avoiding the necessity of expensive instruments, label-free detection, a short time of operation and ease of use [59].

**Figure 8:**
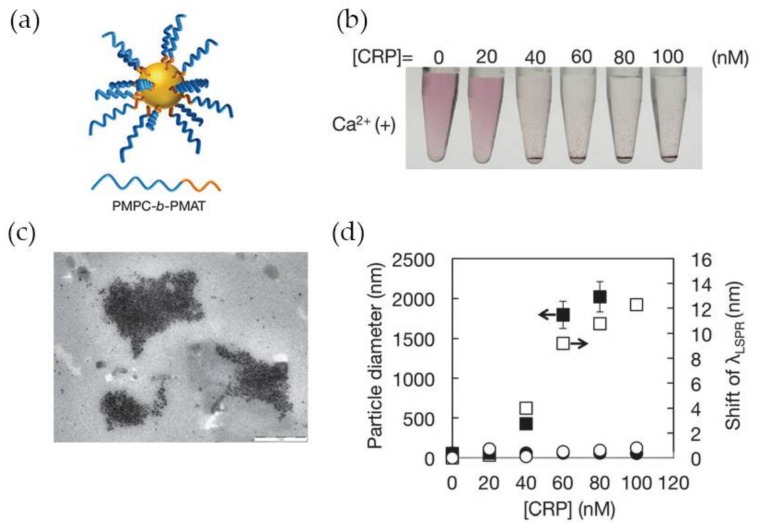
(**a**) Representation of PMPC-b-PMAT-protected Au NPs; (**b**) Photographs of Au NPs in presence of CRP at variable concentration; (**c**) Transmission electron microscope (TEM) image of PMPC-b-PMAT-protected Au NPs after CRP addition; (**d**) Changes in particle diameter (nm) (closed symbol) and λ_LSRP_ shift (open symbol) after addition of CRP without Ca^2+^ (circles) and with Ca^2+^ 1 mM (squares). Reproduced with permission from ref. [59]. Copyright The Royal Society of Chemistry, 2014.

A number of LSPR-based assays for CRP detection have been reported in the last years, namely using gold nanoparticles functionalized with synthetic polymers containing PC-groups in alternative to antibodies for specific recognition of CRP. Previous works have clarified that polymers having in their structure 2-methacryloyloxyethyl phosphorylcholine (MPC) moieties can effectively suppress non-specific binding with other proteins present with CRP [75,76] which makes MPC very attractive for the development of CRP biosensing systems. Iwasaki and co-workers [59] synthetized Au NPs functionalized with a thiol-terminated block copolymer containing PC groups, poly(2-methacryloyloxyethyl phosphorylcholine)-b-poly(*N*-methacryloyl-(l)-tyrosine methylester) (PMPC-*b*-PMAT) (Figure 8a). The PMPC-*b*-PMAT at the surface of Au NPs provided excellent colloidal stability owing to steric stabilization and a dense hydration layer enriched in PC groups. In the presence of Ca^2+^ ions and CRP, a gradual aggregation of protected Au NPs with increasing concentration of CRP (up to 100 nM) was observed (Figure 8b). The aggregation was ascribed to the interaction of CRP with PC groups which led to inter-particle cross-linking of modified Au NPs. The particle aggregation was confirmed by transmission electron microscopy and could be monitored by the shift of λ_LSRP_ (Figure 8c,d, respectively). No aggregation was observed in the absence of Ca^2+^ regardless of CRP concentration (Figure 8d), thus confirming that Ca^2+^ plays a crucial role in the interaction between CRP and PC groups. The detection limit achieved using this system was between 20 and 40 nM. 

Using MPC as well, Kitayama and Takeuchi [67] have prepared poly(MPC)-grafted Au NPs (PMPC-g-Au NPs) by surface-initiated atom transfer radical polymerization for CRP detection. The PMPC-g-Au NPs obtained after 45 h polymerization were highly stable in tris-HCl (10 mM) buffer solution supplemented with NaCl (140 mM) and CaCl_2_ (20 mM) salts, and subsequently tested for CRP sensing. The changes on the LSPR band were assessed by the variation of the *A/D* parameter, as Equation (2) describes:

Δ(*A/D*)/(*A/D*)_0_ = ((*A/D*) − (*A/D*)_0_)/(*A/D*)_0_ (2)

The *A* and *D* parameters stand for the aggregated state (550–700 nm) and the dispersed state (490–540 nm), respectively. These parameters are given by the integral of the Au NPs spectra in the respective spectral zone and where (*A/D*)_0_ was the initial *A/D* parameter, prior to CRP addition. The addition of CRP led to the aggregation of PMPC-g-Au NPs with a consequent increase in the value of Δ(*A/D*)/(*A/D*)_0_. The system was tested at variable concentrations of CRP (10, 100, 1000 μg·mL^−1^), the Δ(*A/D*)/(*A/D*)_0_ value was measured and plotted against CRP concentrations. The detection limit for CRP was around 50 ng·mL^−1^, which is comparable to detection limits achieved using antibody-based CRP sensors [77]. Human serum albumin (HSA) was used as reference protein to confirm the specific binding of the system to CRP. No change was observed on the *A/D* parameter for HSA concentrations up to 100 ng·mL^−1^ which indicated that the PMPC functionalized Au NPs were less inclined for nonspecific binding interactions with other proteins. The authors have demonstrated the successful CRP detection in 1 wt % human serum solution using PMPC-g-Au NPs, at a level of sensitivity sufficient for use in clinical diagnostics as hs-CRP.

**Figure 9:**
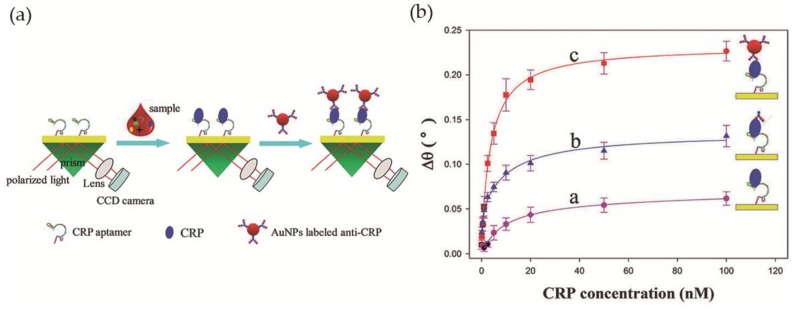
(**a**) Scheme of the biosensor operating with antibody-aptamer sandwich assay; (**b**) Variation of the refractive index with CRP concentration using direct, aptamer-antibody sandwich and Au NP-enhanced aptamer-antibody sandwich measurements. Reproduced with permission from ref. [60]. Copyright The Royal Society of Chemistry, 2016.

Wu et al. [60] developed a simple LSPR-based sensor for CRP detection, with improved sensitivity, based on an aptamer-antibody sandwich assay. The DNA aptamer, with high affinity and specificity towards CRP, was immobilized on the surface of the sensing region of the sensor, a bare gold chip, while Au NPs were bioconjugated with anti-CRP and used for signal amplification. In the presence of the analyte CRP, the immobilized aptamers retain CRP proteins on the Au film by specific binding. After signal enhancement with the addition of anti-CRP coated Au NPs, the resonance angle was recorded (Figure 9a). The relation between the variation of the resonance angle and the CRP concentration was investigated using three different strategies: direct measurement, aptamer-antibody sandwich measurement, and Au NP-enhanced aptamer-antibody sandwich measurement. The latter provided the best results with the lowest detection limit, 10 pM, in diluted human serum (Figure 9b). In addition, the selectivity of the system towards CRP was tested in the presence of five proteins—immunoglobulin G, hemoglobin, human serum albumin, transferrin and myoglobin—and good selectivity for CRP was observed.

**Figure 10:**
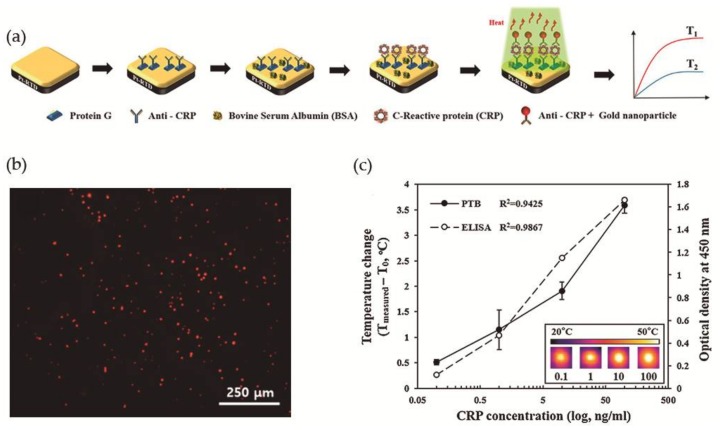
(**a**) Schematic illustration of the construction of the photothermal biosensor; (**b**) Fluorescence microscopy image of the biosensor in presence of CRP labeled with a fluorescent dye; (**c**) Evaluation of PTB and ELISA in the detection of different concentrations of CRP. Reproduced with permission from ref. [78]. Copyright Elsevier B. V., 2017.

More recently, Lee et al. [78] took advantage of the photothermal property of Au NPs, i.e., the ability to generate heat upon exposure to light at a specific wavelength, for the development of a photothermal biosensor (PTB) to measure CRP concentrations in human saliva. The PTB was prepared by a conventional photolithography process and was composed of a platinum-resistive temperature detector (Pt-RTD sensor) coated with a silicon dioxide layer and at the top a gold layer as sensing region, as illustrated in Figure 10a. The Pt-RTD sensor measured electric resistance changes which are linear against temperature changes promoted by the photothermal effect. Monoclonal antibodies for CRP (anti-CRP) were immobilized on the gold layer of the sensing region using Protein G conjugated with a fluorescent dye and the free surface was then blocked with BSA to avoid non-specific binding interactions. In a typical CRP detection assay, a CRP solution was added to the sensing region and washed with PBS for the removal of unreacted CRP. Spherical gold nanoparticles of 30 nm diameter and conjugated with polyclonal anti-CRP (Au NPs-conjugated anti-CRP) were then added to perform the sandwich immunoassay and allowed to react (Figure 10b). Then the sensing region was irradiated (λ = 532 nm) to induce the photothermal effect and the temperature increment was measured. A correlation coefficient of 0.9425 was found between the CRP concentration and temperature increment (Figure 10c). A major advantage that was found was that the biosensor could be reused after washing with a surfactant solution for the removal of Au NPs. Nevertheless, to avoid protein denaturation the temperature of the system could not be superior to 40 °C, which limited the laser power to 10 mW and the irradiation time to a maximum of 90 s, and the optimal Au NPs concentration was 1.8 × 10^12^ N·mL^−1^. 

Aiming to compare the efficiency of this system, authors have performed a commercial ELISA biodetection test for the same CRP concentration range and achieved a correlation coefficient of 0.9867, which is larger than the coefficient obtained using the PTB (Figure 10c). However, PTB had the advantages that it only needed 7 μL of the sample for analysis and that CRP detection linear range was 0.1 to 100 ng·mL^−1^ with a detection limit of 0.1 ng·mL^−1^. In addition, a successful human saliva test from 5 healthy individuals was performed, where the samples were not filtered or diluted.

### 3.3. Au NP-Based Point-of-Care (POC) Sensors for CRP Detection

POC diagnostics are designed to provide fast and simple measurements and reliable results to allow clinicians to make quick medical decisions, without the need of expensive equipment or skilled technicians. Portability and low cost are also important requirements of POC tests, in order to be accessible in resource limited settings. For example, the lateral-flow assay (LFA) is an important POC diagnostic format that uses paper-based test strips, commercially available for the rapid diagnosis of several health conditions (e.g., pregnancy) and several biomarkers [79,80], including CRP. Owing to their unique properties Au NPs can be employed to develop inexpensive paper-based POC tests, with facile colorimetric, spectrometric or electrochemical detection [17]. The high optical extinction coefficient of Au NPs, which is more than 1000 times higher than in organic dyes [81] offers the possibility of high-sensitivity colorimetric detection in a POC platform. However, the combination of immunoassays with Au NPs as readout is still a challenge.

**Figure 11:**
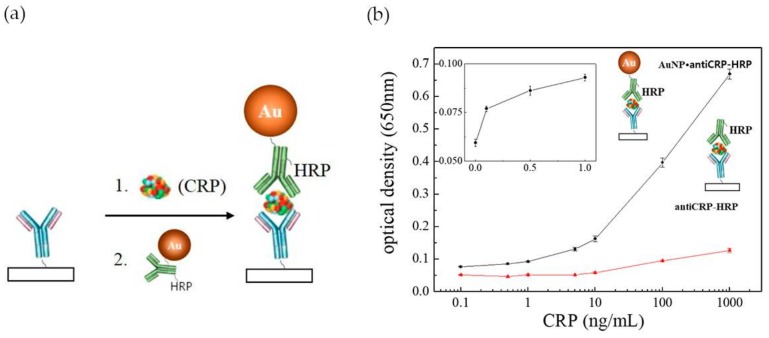
(**a**) Schematic illustration of the use of Au NPs conjugated with HRP-labelled anti-CRP on “capillary ELISA”. Reproduced with permission from ref. [54]. Copyright Elsevier, 2016; (**b**) Representation of optical density against CRP concentrations plot in Au NP “capillary ELISA” (black line) and common ELISA (red line). The inset represents the linear plot of lower CRP concentrations range in Au NP “capillary ELISA”. Reproduced with permission from ref. [83]. Copyright MPDI, 2017.

In the last years a number of innovative POC tests have been proposed either by academia research or as commercial products [80,82]. However, POC assays are still limited in terms of sensitivity and quantitation, when compared to laboratory-based diagnosis, which constrains their application. Commercially available LFA assays (Cholestech (Hayward, CA, USA), Milenia Biotec (Gießen, Germany), BÜHLMANN (Schönenbuch, Switzerland)) are unable to detect CRP concentrations below 0.2 mg·L^−1^ [83]. Efforts have been performed to enhance the performance of LFA tests in CRP detection, by improving the configuration of ELISA based assays (e.g., “capillary ELISA” [54], three-line LFA [13] and 3 test-line assay [55]) incorporating tailored size functionalized Au NPs [84] or using a combined approach [83]. “Capillary ELISA” assays performed with Au NPs 10 nm in size conjugated with Horseradish Peroxidase (HRP)-labelled anti-C reactive protein (anti-CRP) were faster and more sensitive than conventional ELISA assay, allowing to detect 0.1 ng·mL^−1^ CRP after 30 s incubation (Figure 11a). By contrast, conventional ELISA barely recognized 10 ng·mL^−1^ of CRP (Figure 11b) [83]. This excellent performance was ascribed to a synergistic effect between Au NPs and the capillary system. Byova et al. [55] have developed an immunochromatographic test for the rapid and simultaneous detection of three diagnostically important proteins that reflect cardiac dysfunction, troponin I (TnI), fatty acid binding protein (FABP) and CRP. The assay comprised three test lines, one for each protein, and the formation of the immuno-complexes between antibodies labelled with gold nanoparticles and the distinct biomarkers. The detection limit for CRP was 600 ng·mL^−1^, in a linear range of 1000–15,000 ng·mL^−1^. Overall, the results of this multiparametric assay were in agreement with those obtained by ELISA.

**Figure 12:**
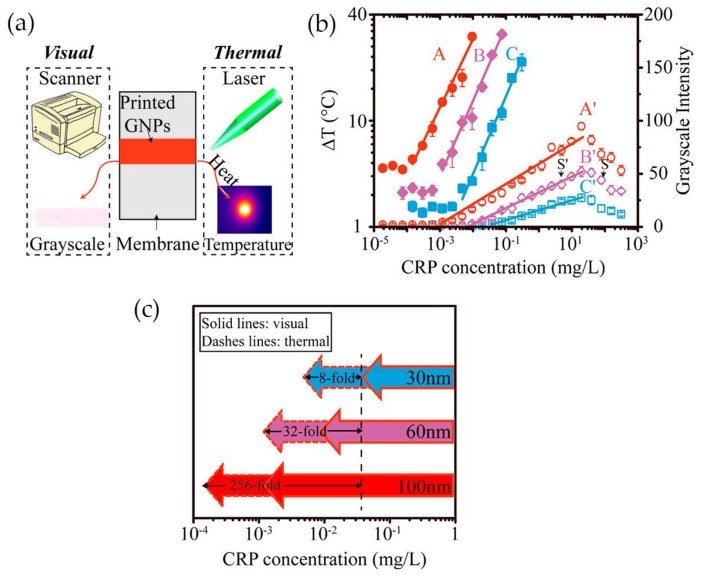
(**a**) The two methods used for evaluate CRP detection: visual and thermal; (**b**) Illustration of the correlation between temperature increment and grayscale intensity with CRP concentration; (**c**) Representation of thermal and visual detection of CRP on LFA, using Au NPs with 30, 60 and 100 nm of average size. Reproduced with permission from ref. [84]. Copyright American Chemical Society, 2017.

Having in mind the photothermal effect of gold nanoparticles, Zhan and co-workers [84] have developed an LFA with CRP detection based both on optical and thermal contrast signals (Figure 12a). The readout was done using a thermal contrast amplification reader that collected the temperature changes of gold nanoparticles upon laser irradiation in the test line [85]. The temperature increment was correlated with the concentration of the analyte (Figure 12b). Using 30 nm Au NPs, thermal contrast analytical sensitivity was enhanced 8-fold over classical optical detection LFA (Figure 12c). The authors demonstrated via modeling that the size of gold nanoparticles is a major parameter affecting the analytical performance of the LFA, with influence on the bioconjugated surface area for CRP detection and optical and thermal signals intensity. Model results have shown that larger-size gold nanoparticles have higher binding affinity and are detected at lower concentration owing to stronger light absorption and scattering properties, thus improving detection. Ultimately, using 100 nm size Au NPs, the CRP detection limit could be improved 256-fold over conventional LFA with 30 nm Au NPs (Figure 12c). However, larger particles, despite achieving higher sensitivity, can increase chances of non-specific interactions and block the pores of the paper-based substrate, with consequences for detection accuracy.

## 4. Conclusions

Covalent immobilization of antibodies onto the surface of gold nanoparticles still prevails in the development of assays for CRP detection. In agreement, the majority of the assays are derived from the classical ELISA. Yet, some innovative aspects have been explored in the design of the assays, such as the photothermal effect of Au NPs and the construction of a “capillary ELISA”. Among the several assays for CRP detection, the best limit of detection (LOD) (0.33 fg·mL^−1^) was reached using a complex system involving the covalent modification of Au NPs and Cu NPs intercalated with DNA concatemers attached to a secondary antibody. This approach is far from being economically viable and efforts should be devoted to the design of simpler strategies for CRP detection and quantification.

The most sensitive LFA was achieved using antibodies against CRP (pg·mL^−1^). Thus, despite the disadvantages, antibodies are able to help accomplish a good LOD when compared with strategies using Au NPs functionalized with PC groups (LOD 0.23–0.47 ng·mL^−1^ [59], 50 ng·mL^−1^ [67]) or aptamers (1.2 ng·mL^−1^ [60], 0.0017 ng·mL^−1^ [56]). Further studies are essential to develop portable POC assays with enhanced LOD down to pg·mL^−1^.

## Figures and Tables

**Table 1:** Strategies for surface modification and bioconjugation of gold nanoparticles (Au NPs) aiming for specificity towards C-reactive protein (CRP).

Type	Strategy	Attached Molecule	Reference
Covalent	Surface functionalization with –COOH (thiolated acids). Linkage to aminated molecules via carbodiimide chemistry	O-phosphorylethanolamine (PEA)	[58]
		Antibody	[57]
	Formation of Au-S bond	Thiolated aptamer	[56]
		Poly(2-methacryloyloxyethylphosphorylcholine) (PMPC)	[67]
		Poly(2-methacryloyloxyethyl phosphorylcholine)-b-poly(*N*-methacryloyl-(l)-tyrosine methylester) (PMPC-b-PMAT)	[59]
Non-covalent	Coating (electrostatic/hydrophobic interactions with Au surface)	PEA	[68]
		Antibody	[13,54,56]

**Table 2:** Summary of most representative assays for CRP detection based on Au NPs. Information is provided regarding the linear range of CRP detection (ng·mL^−1^), the interfering protein tested and the limit of detection (ng·mL^−1^).

Type of Assay	Description	Interferent Tested Proteins	Linear Range (ng·mL^−1^)	Limit of Detection (ng·mL^−1^)	Publication Year	Reference
**Electrochemical detection**	Au NPs functionalized with MPA (Au–S bond) and linked to Ab-αCRP using EDC/NHS. Au NPs were used to enhance conductivity.	IgG	0.01–10	19.38	2014	[12]
	rGO-Au NPs hybrid structures. Au NPs generated in situ, functionalized with MPA (Au–S bond) and linked to anti-CRP using *N*-(3-Dimethylaminopropyl)-*N*′-ethylcarbodiimide (EDC)/*N*-Hydroxysuccinimide (NHS). Au NPs were used to enhance conductivity.	BSA	0.001-2 ^1^	0.08	2015	[57]
	MoS_2_/PANI nanocomposite decorated with Au NPs nanoparticles. Non-covalent immobilization of anti-CRP.	BSA, Glu, HCG, Gly	0.2–80	4 × 10^−4^	2016	[18]
	Modified sandwich immunoassay using Au NPs for antibody attachment and Cu NPs to generate the electrochemical signal.	AFP, CEA, l-Cys, Lysine and UA	10 × 10^−6^–70	0.33 × 10^−6^	2017	[74]
	Silica microspheres decorated with Au NPs functionalized with anti-CRP for CRP detection and for signal amplification.	BSA, AFP, PSA, CEA, Cys, Glu, UA and Lys	0.005–125	0.0017	2017	[56]
**Localized surface plasmon resonance (LSPR)-based detection**	Modification of Au NPs surface with PMPC-*b*-PMAT, containing PC groups for specific interaction with CRP.	-	0–0.95	0.23–0.47	2014	[59]
	Au NPs were modified with PMPC, containing PC groups for specific interaction with CRP.	HSA	-	50	2014	[67]
	Aptamer-antibody sandwich assay. DNA aptamer immobilized in a gold chip. Au NPs modified with anti-CRP.	HSA, TRF, Myo, Hb and IgG	-	1.2	2016	[60]
	Photothermal biosensor. Au NPs conjugated with anti-CRP in a sandwich immunoassay.	BSA	0.1–100	0.1	2017	[78]
**Point-of-care (POC) sensors (portable)**	Au NPs conjugated with anti-CRP. An antigen line was added to the conventional two-line lateral-flow assay (LFA) sensor, for detecting CRP within a broad concentration range.	BSA, IgG (control line)	0.5–1	0.65	2014	[13]
	Au NPs conjugated with antibodies against CRP, troponin I (TnI) and fatty acid binding protein (FABP) (3 test-line assay)	BSA, IgG (control line)	1000–15,000	600	2016	[55]
	Au NPs were utilized for prove the efficiency of the amination strategy used for CRP detection via vapor-phase.	BSA	-	1	2016	[54]
	Au NPs were conjugated with anti-CRP-HRP and used in a “capillary enzyme-linked immunosorbent assay (ELISA)”.	-	-	0.1	2017	[83]
